# The Imbalance of Circulating Follicular T Helper Cell Subsets in Primary Sjögren’s Syndrome Associates With Serological Alterations and Abnormal B-Cell Distribution

**DOI:** 10.3389/fimmu.2021.639975

**Published:** 2021-03-19

**Authors:** Krisztina Szabó, Ilona Jámbor, Antónia Szántó, Ildikó Fanny Horváth, Tünde Tarr, Britt Nakken, Peter Szodoray, Gábor Papp

**Affiliations:** ^1^ Division of Clinical Immunology, Faculty of Medicine, University of Debrecen, Debrecen, Hungary; ^2^ Department of Immunology, Oslo University Hospital, Rikshospitalet, Oslo, Norway

**Keywords:** follicular T helper cell, follicular regulatory T cell, B cell, chemokine receptors, primary Sjögren’s syndrome

## Abstract

Since B-cell hyperactivity and pathologic antibody response are key features in the immunopathogenesis of primary Sjögren’s syndrome (pSS), the role of follicular T helper (T_FH_) cells as efficient helpers in the survival and differentiation of B cells has emerged. Our aim was to investigate whether a change in the balance of circulating (c)T_FH_ subsets and follicular regulatory T (T_FR_) cells could affect the distribution of B cells in pSS. Peripheral blood of 38 pSS patients and 27 healthy controls was assessed for the frequencies of cT_FH_ cell subsets, T_FR_ cells, and certain B cell subpopulations by multicolor flow cytometry. Serological parameters, including anti-SSA, anti-SSB autoantibodies, immunoglobulin, and immune complex titers were determined as part of the routine diagnostic evaluation. Patients with pSS showed a significant increase in activated cT_FH_ cell proportions, which was associated with serological results. Frequencies of cT_FH_ subsets were unchanged in pSS patients compared to healthy controls. The percentages and number of cT_FR_ cells exhibited a significant increase in autoantibody positive patients compared to patients with seronegative pSS. The proportions of transitional and naïve B cells were significantly increased, whereas subsets of memory B cells were significantly decreased and correlated with autoantibody production. Functional analysis revealed that the simultaneous blockade of cT_FH_ and B cell interaction with anti-IL-21 and anti-CD40 antibodies decreased the production of IgM and IgG. Imbalance in T_FH_ subsets and T_FR_ cells indicates an ongoing over-activated humoral immune response, which contributes to the characteristic serological manifestations and the pathogenesis of pSS.

## Introduction

Primary Sjögren’s syndrome (pSS) is a chronic autoimmune disease displaying slow progression and affecting primarily middle-aged women nine-times more frequently than men. Histologically, the hallmark feature of pSS is the periductal cellular infiltration of lacrimal and salivary glands resulting in the damage and dysfunction of glandular tissue. Clinically, patients suffer from mouth dryness (xerostomia) and eye dryness (xerophtalmia) predominantly; however, various exocrinopathic symptoms are also frequently present. The clinical manifestations in pSS are not homogenous, besides glandular symptoms; the disease can involve any organ system and present with a wide variety of extraglandular manifestations affecting skin, genitourinary tract, kidneys, cardiovascular, nervous or musculoskeletal systems (1).

The development of pSS is still unclear; however, recent lines of evidence support a complex network between salivary gland epithelial cells and the innate and acquired immune systems. Epithelial cells, besides being the target of extrinsic and intrinsic factors that cause disruption of glandular tissue integrity, are also active participants in the initiation and perpetuation of the inflammatory process, through the expression of activation markers and toll-like receptors. The upregulation of adhesion and chemokine molecules, along with metabolic stress induced apoptosis initiates the recruitment of innate immune cells and triggers an interferon-related inflammatory cascade (1). Type I interferon production by plasmacytoid dendritic cells induces infiltrating leukocytes and epithelial cells to secrete B-cell activating factor (BAFF), which promotes B-cell survival and not only contributes to B-cell hyperactivity, but also acts as a mediator between innate and adaptive immunity (2, 3). Local accumulation and sustained survival of autoreactive B cells promote the development of ectopic germinal centers (GCs), which establish the perfect niche for autoantibody production or lymphomagenesis (4, 5).

T cell support for B cells is a pivotal mechanism for optimal B-cell selection, development, and activation. A subset of CD4^+^ T cells, termed follicular T helper (T_FH_) cells, are responsible for T-cell dependent humoral response in the B-cell follicles of secondary lymphoid tissues. The cognate interaction between T_FH_ and activated naïve B cells is required for the generation of short-lived plasma cells at the extrafollicular foci as well as the development of long-lived high-affinity, antibody-secreting plasma cells during GC response (6). The ability to differentiate into GC B cells and GC T_FH_ cells depends on antigen-specific contact between these cells and the successful delivery of mutual signals from the very first stage of their interaction (7). T-cell-dependent B-cell response proceeds in three spatiotemporal phases in the lymphoid tissues, including extrafollicular, follicular, and GC reaction phase, which demands the expression of several molecules. B cells in GCs receive help through CD40 ligation (CD40L), inducible T cell costimulator (ICOS) expression as well as *via* cytokine signals, especially interleukin-(IL)-21, which promotes B-cell differentiation. Stable connection between T_FH_ and B cells is essential to prolong cell–cell contact and the effective delivery of help, which is established by signaling lymphocyte activation molecule associated adaptor protein (SAP) (8, 9). In the GCs, B cells undergo repetitive cycles of somatic hypermutation, clonal proliferation, and selection with the specialized aid from T_FH_ cells to ensure the development of memory B cells and high-affinity antibody-secreting plasma cells (10). Whereas the evolution of GCs provides potent immune protection against foreign antigens, the tight regulation of T_FH_ and B-cell interaction is essential to maintain immunological self-tolerance to prevent autoreactivity (11). Regarding the origin of circulating (c) T_FH_ cells, strong evidence is supporting that T_FH_ cells can be transported to neighboring GCs and migrate to the periphery. Activated T cells with decreased expression of characteristic T_FH_ cell markers appear in the blood, and they have the potential to form memory cells (9). These circulating CD4^+^ C-X-C chemokine receptor 5 (CXCR5)^+^ memory T_FH_ cells share functional properties with GC T_FH_ cells and express ICOS and programmed cell death protein 1 (PD-1) molecules, however, in a tempered level. Human blood memory T_FH_ cells form a heterogeneous group and include several subsets with different phenotypes and functions according to the presence of ICOS, PD-1, C-C chemokine receptor type 7 (CCR7), CD62L, and chemokine receptors CXCR3, CCR6 (12, 13). The first set of parameters distinguishes CXCR5^+^CCR7^−^CD62L^−^ effector memory and CXCR5^+^CCR7^+^CD62L^+^ central memory cT_FH_ cells. The expression of the two molecules is probably promoting the migration of the cells to secondary lymphoid tissues. Memory cell types can be further divided into ICOS^−^PD-1^+^CCR7^int^ and ICOS^−^PD-1^−^CCR7^hi^ quiescent and ICOS^+^PD-1^++^CCR7^lo^ activated memory cT_FH_ cells. The second set of parameters defines CXCR3^+^CCR6^−^ Th1-like cells (cT_FH_1), CXCR3^−^CCR6^−^ Th2-like (cT_FH_2) and CXCR3^−^CCR6^+^ Th17-like (cT_FH_17) cells (14). Concerning their distinct functional roles, T_FH_2 and T_FH_17 cells are declared as efficient helpers since they induce naïve B cell differentiation into plasma cells and produce immunoglobulin (Ig)A, IgG, and its subclasses. On the other hand, T_FH_1 cells are not competent naïve B-cell helpers; the only exception is when this subset is in an activated state (ICOS^+^PD-1^++^), but their helper capacity is limited to memory B cells (15–18). Recently, a subpopulation of regulatory T cells expressing Foxp3 and CXCR5, called follicular regulatory T (T_FR_) cells, was identified, which have a capability to modify GC responses in several ways: direct regulation of T_FH_-cell proliferation *via* the interruption of costimulation or due to the modulation of metabolic pathways in T_FH_ and B cells (19). Consequently, investigations on T_FH_ and B cell interactions in pSS not only can shed light on disease pathogenesis, but can also show the potential use of T_FH_ cells as a biomarker or therapeutic target in autoimmune diseases as an attractive possibility.

In our study, we investigated the disproportion of T_FR_ and cT_FH_ cell subsets and assessed their correlations with disease activity and with various circulating B cell subpopulations in patients with pSS and healthy individuals. Moreover, we evaluated the role of cT_FH_ cells in helping B cells by an *in vitro* functional assay.

## Materials and Methods

### Subjects

Based on our clinical practice, patients underwent evaluation according to the American-European Consensus Group (AECG), and the diagnosis of pSS was based on it (20). The diagnosis of patients was also revised in agreement with the 2016 American College of Rheumatology (ACR)/European League Against Rheumatism (EULAR) criteria for pSS (21). A total of 38 patients with pSS (37 female and one male; median age: 54 (range 37–74) years) fulfilling both classification criteria were included in the present study. Patients were diagnosed at the Division of Clinical Immunology, University of Debrecen, where they received regular follow-up treatment. The median disease duration was 12 (range 1–25) years. Since immunosuppressive and immunomodulatory drugs fundamentally affect the functions and numbers of the investigated lymphocytes, pSS patients with ongoing immunomodulatory treatments were excluded from the study. Systemic disease activity in pSS was assessed with the EULAR Sjögren’s Syndrome Disease Activity Index (ESSDAI) at the time of the laboratory examinations (22). The mean ESSDAI was 2.44 (± 0.51, range 1–4), which indicated currently a low disease activity without the necessity of ongoing immunosuppressive drug administration. During the total disease course, 23 patients suffered from systemic manifestations, such as vasculitis, Raynaud’s phenomenon, polyarthralgia, and polyneuropathia, while 15 patients had common glandular involvements. Based on routine laboratory results, 24 patients were positive for anti-Ro/SSA autoantibody (SSA >10 U/ml); moreover, 14 of them showed anti-La/SSB seropositivity as well. Twenty-nine age and sex-matched healthy volunteers (27 female and two male; median age: 46 (36–57) years) served as controls. The characteristics of pSS patients and healthy participants are presented in **Table 1**. The study participants diagnosed with viral or bacterial infections were excluded. Informed written consent was obtained from all patients. The study has been approved by the Ethics Committee of our University (protocol number: 4879-2017) and the Policy Administration Services of Public Health of the Government Office (protocol number: 1660-4/2018). All data stored and all experiments carried out were in compliance with the Declaration of Helsinki.

**Table 1:** Demographic characteristic of patients with pSS and healthy controls.

	pSS (n = 38)	Control (n = 29)
Age, years (range)	54 (37–74)	46 (36–57)
Female sex, n (%)	37 (97.4)	27 (93.1)
Systemic involvement, n (%)	23 (60.5)	NA
ESSDAI, mean [SD (min-max)]	2.44 [± 0.51 (1–4)]	
Anti-Ro/SSA positivity, n (%)	24 (63.2)	NA
Anti-Ro/SSA titer, U/ml	100.0 (99.7–100.0)	NA
Anti-La/SSB positivity, n (%)	14 (36.8)	NA
Anti-LA/SSB titer, U/ml	89.9 (86.2–98.4)	NA
RF, n (%)	29 (76.3)	NA
RF titer, IU/ml	24 (16.5–71.5)	NA
IC titer, extinction	81.5 (56.0–150.8)	NA
IgG titer, g/L	16.28 ± 7.84	NA
IgA titer, g/L	2.80 ± 1.56	NA
IgM titer, g/L	1.33 ± 0.91	NA
Lymphocyte count, ×10^9^/L	1.52 (1.23–1.84)	1.86 (1.39–2.41)

Data are displayed as mean ± SD or median (25th–75th percentiles). NA, no data available; pSS, primary Sjögren’s syndrome; ESSDAI, EULAR Sjögren’s Syndrome Disease Activity Index; RF, rheumatoid factor; IC, immune complex; Ig, immunoglobulin.

### Cell Surface Staining and Flow Cytometric Analysis of T_FH_ and B Cell Subsets in Peripheral Blood Mononuclear Cells

Immunophenotyping of lymphocytes was performed by multicolor flow cytometry. Venous blood was collected in vacutainer heparin tubes, and peripheral blood mononuclear cells (PBMCs) were isolated by Ficoll-Histopaque (Sigma-Aldrich, St Louis, MO, USA) density-gradient centrifugation. Harvested cells were washed twice and stained with anti-IgD-fluorescein isothiocyanate (FITC) (clone: IADB6, Beckman Coulter Inc, Fullerton, CA, USA), anti-CD27-phycoerythrin (PE) (clone: 1A4CD27, Beckman Coulter), anti-CD19-phycoerythrin-Cyanine dye 5 (PE-Cy5) (clone: J3-119, Beckman Coulter), anti-CD38-FITC (clone: HIT2, BioLegend, San Diego, CA, USA), anti-CD24-allophycocyanin (APC) (clone: ML5, BioLegend), anti-CXCR5-Alexa Fluor 488 (clone: RF8B2, BD Biosciences, San Diego, CA, USA), anti-ICOS-PE (clone: DX29, BD Biosciences), anti-PD-1-Peridinin-chlorophyll protein-Cyanine dye 5.5 (PerCP-Cy5.5) (clone: EH12.1, BD Biosciences) and anti-CD4-APC (clone: RPA-T4, BioLegend) monoclonal antibodies. Cell staining was carried out for 20 min at 4°C in the dark, then washed twice, and prepared for measurements. In case of B cells, at least 10.000 CD19^+^ events were analyzed, while T_FH_ cell data were collected from at least 75.000 CD4^+^ events per sample within the whole lymphocyte population.

**Figure 1:**
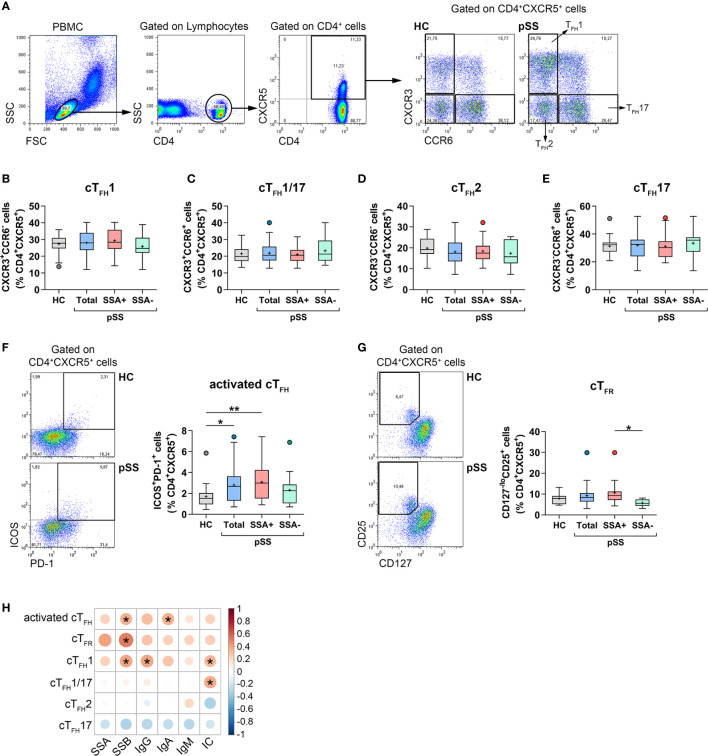
The distribution of cT_FH_ subsets and T_FR_ cells in patients with pSS and their association with laboratory serological markers. PBMCs were isolated from 38 pSS patients and 29 healthy controls (HC) then were stained with fluorochrome-labeled antibodies as described previously. Peripheral circulating (c)T_FH_ subsets were quantified as their percentage within CD4^+^CXCR5^+^ lymphocyte population. **(A)** Representative dot plots show the gating strategy and the division of T_FH_ cell subpopulations. Percentages of cT_FH_1 **(B)**, cT_FH_1/17 **(C)**, cT_FH_2 **(D)**, cT_FH_17 **(E)** in pSS patients (n = 38), pSS without anti-SSA(−) (n = 14), pSS with anti-SSA(+) (n = 24) and HCs (n = 29). **(F)** Representative dot plots show the gating of ICOS^+^PD-1^+^ activated cT_FH_ cells in pSS and HC. Percentages of activated cT_FH_ cells. **(G)** Representative dot plots display the gating of T_FR_ cells in pSS and HC. Frequencies of T_FR_ cells. **(H)** Correlation analysis of the percentages of T_FH_ cell subsets and T_FR_ cells with different serological parameters in patients with pSS. One-way ANOVA with Tukey’s multiple comparisons test **(B, D, E)** or Kruskal–Wallis test with Dunn’s multiple comparisons test **(C, F, G)** was used. Correlation analysis was performed using Spearman’s test **(H)**. Box plots represent the interquartile range (IQR) with a line in the middle as median and “+” sign as the mean value. Statistically significant differences are indicated by *p < 0.05; **p < 0.01.

As described by Morita et al., three T_FH_ subsets could be identified according to the expression of CXCR3 and CCR6 chemokines (15). Based on this study, Th1-like T_FH_1 (CXCR3^+^CCR6^−^), T_FH_1/17 (CXCR3^+^CCR6^+^), Th2-like T_FH_2 (CXCR3^−^CCR6^−^) or Th17-like T_FH_17 (CXCR3^−^CCR6^+^) cells were defined in CD4^+^CXCR5^+^ blood T cells with the help of anti-CXCR3-PE (clone: G025H7, BioLegend) and anti-CCR6-PerCP-Cy5.5 (clone: G034E3, BioLegend) monoclonal antibodies. For the determination of T_FR_ cells we used anti-CD127-PE (clone: R34.34) and anti-CD25-PE-Cy5 (clone: B1.49.9) (both from Beckman Coulter) monoclonal antibodies besides CD4 and CXCR5. Fluorescence Minus One (FMO) controls were used to determine gate settings in multicolor panels. The flow cytometric examination was performed with a FACS Calibur instrument (Becton Dickinson, Franklin Lakes, NJ, USA). Data were analyzed using FlowJo Software (Treestar, Ashland, OR, USA). Fig. 1A demonstrates the gating strategy and the division of T_FH_ cell subpopulations. Cell viability was assessed in a number of fresh samples using 7-Aminoactinomycin D (7-AAD) staining. The percentages of non-viable cells were 3.69 ± 0.73 in total PBMCs, 0.13 ± 0.10 in CD4^+^ lymphocytes, and 0.03 ± 0.02 in case of CD19^+^ lymphocytes (**Supplementary Figure 1C**).

### Cell Separation, Cell Culture and Functional Analysis

B cells were magnetically isolated from the PBMCs using CD19^+^ B cell isolation kit (Miltenyi Biotec; Bergisch Gladbach, Germany). CD4^+^ T cells were enriched from PBMCs using CD4^+^ T cell isolation kit, then CXCR5^−^ and CXCR5^+^ cell populations were separated within these cells with the application of CD185 (CXCR5)-biotin antibody and anti-biotin microbead (all from Miltenyi Biotec) according to the manufacturer’s instructions (healthy controls, n = 7; pSS patients, n = 5). Cell purity was assessed by flow cytometry: it was greater than 98% for B cells, while the purity was above 80% for cT_FH_ cells. B cells were pre-activated by cross-linking of B-cell receptor (BCR) with 2.5 μg/ml affinity purified F(ab′)2 fragment of goat anti-human IgG + IgM (H + L) (Jackson ImmunoResearch, Baltimore, PA, USA) in the presence of recombinant human IL-2 and IL-10 proteins (both 50 ng/ml) (PeproTech, Cranbury, NJ, USA) for 24 h at 37°C in 5% CO_2_ milieu in Roswell Park Memorial Institute (RPMI) 1640 medium supplemented with 10% heat-inactivated fetal bovine serum in 96-well flat-bottom plates. For *in vitro* co-culture assay, 5 × 10^4^ autologous B cells were cultured with 5 × 10^4^ CD4^+^CXCR5^−^ T cells or 5 × 10^4^ CD4^+^CXCR5^+^ cT_FH_ cell in the presence of staphylococcal enterotoxin B (SEB) (Sigma-Aldrich; Steinheim, Germany) (1 μg/ml) in 96-well U-bottom plates. Blocking of T–B cell interaction was performed using 3 μg/ml anti-human IL-21 neutralizing antibody (clone MT216G/21.3m, MabTech Ab) and 5 μg/ml anti-human CD40/TNFRSF5 (clone 82102; R&D Systems, MN, USA). At day 7, supernatants were collected and stored at −70°C until further measurements, while cells were harvested then stained with the combination of anti-CD38-FITC, anti-CD27-PE, anti-CD4-APC monoclonal antibodies and 7-AAD viability solution. Plasmablasts were assessed using FACS Calibur flow cytometer and further analyzed with FlowJo software.

### Enzyme Linked Immunosorbent Assay

IgG and IgM concentrations were measured in the supernatant of the co-culture by enzyme-linked immunosorbent assay (ELISA) kits according to the manufacturer’s protocols (MabTech AB, Nacka Strand, Sweden).

### Assessment of Routine Laboratory Parameters

All patients were followed up on a routine basis, and their records contain detailed information on symptoms, clinical conditions, laboratory, and other findings of each visit. Routine laboratory measurements were carried out at the Department of Laboratory Medicine, Faculty of Medicine, University of Debrecen. Blood cell counts including total lymphocyte counts were analyzed with ADVIA 2120i hematology system (Siemens, Munich, Germany). Autoantibody levels were determined by enzyme-linked immunosorbent assay (ELISA) with AUTOSTAT II kits (Hycor Bio-medical, Indianapolis, IN, USA), according to the manufacturer’s instructions (anti-Ro/SSA; normal: ≤10 U/ml and anti-La/SSB; normal: ≤10 U/ml). Serum Ig concentrations were analyzed by turbidimetric method (Dialab GmbH, Wiener Neudorf, Austria). Rheumatoid factor (RF; normal: ≤20 IU/ml) was measured by quantitative nephelometry (Dialab). Circulating immune complexes (ICs) were detected by polyethylene glycol precipitation method. Laboratory reference range was an extinction of 0 to 170 for IC. The results were given according to the analytical measurement range stated in the manufacturers’ instructions.

### Statistical Analysis

Data and statistical analyses were performed with GraphPad Prism 8 software (GraphPad Software, San Diego, USA). Descriptive data was represented in box plots of interquartile range (IQR) with a line in the middle as median and “+” sign as the mean value. Tukey whiskers show the 25th percentile minus 1.5IQR and 75th percentile plus 1.5IQR. Functional assay data displayed in scattered dot plots and bars with mean ± standard deviation. Kolmogorov–Smirnov and Shapiro–Wilk normality tests were used to assess normal distribution. In cases of normal distribution, one-way analysis of variance (ANOVA) with Tukey’s *post hoc* test for pairwise multiple comparisons was carried out. When data sets deviated from normality, Kruskal–Wallis test followed by Dunn’s *post hoc* test for pairwise multiple comparison was used. Two-way ANOVA with posttest Tukey’s test or Bonferroni’s test for multiple comparisons was used to assess data in *in vitro* experiments. Statistics and visualization of the correlation matrices were done with the R 3.4.0 software, using the Hmisc and Corrplot packages (23–25). Spearman’s correlation analysis was used for the correlations between the percentages of cell subsets and serological parameters, and R-values were obtained using Corrplot function. Differences were considered statistically significant at *p ≤* 0.05.

## Results

### Changes in the Proportions of Circulating T_FH_ Cell Subsets and T_FR_ Cells in Patients With pSS

We previously described elevated percentages of circulating T_FH_ cells in pSS (26). In order to confirm our former observation, we determined cT_FH_ cell frequencies in a significantly larger patient cohort with well characterized B-cell abnormalities. Additionally, we further characterized blood T_FH_ cell subsets in these patients. Lymphocytes were gated based on a forward and side scatter plot followed by a CD4^+^ cell gate. The percentages of T_FH_ cell subsets, including T_FH_1, T_FH_2, T_FH_17, and T_FH_1/17 were quantified within the CD4^+^CXCR5^+^ lymphocytes of peripheral blood (**Figure 1A**). Several studies have shown that cT_FH_ cells belong to memory CD45RA^−^CD4^+^ T cells (15). To confirm the credibility of our protocol, we evaluated the relative fluorescence expression of CD45RA on circulating CD4^+^CXCR5^+^ as well as CD4^+^CXCR5^+^PD-1^+^ T cells and reinforced that both subsets barely expressed CD45RA compared to CXCR5^−^ T cells; thus they resembled memory cells (**Supplementary Figure 2B**). To assess whether cT_FH_ subsets were altered in pSS, CXCR3^+^CCR6^−^ T_FH_1, CXCR3^+^CCR6^+^ T_FH_1/17, CXCR3^−^CCR6^−^ T_FH_2, and CXCR3^−^CCR6^+^ T_FH_17 cells were identified and quantified. There was no detectable difference in percentages of each T_FH_ subsets in pSS patients in comparison to controls or in either designated group within pSS (**Figures 1B–E**). Interestingly, the entire group of pSS patients (*p* = 0.0248) and patients with anti-Ro/SSA positivity (*p* = 0.0047) showed a significantly increased frequency of activated ICOS^+^PD-1^+^ cT_FH_ cells, compared to healthy individuals (**Figure 1F**).

Blood T_FR_ cells were identified as CD127^−^CD25^bright^ cells within the CD4^+^CXCR5^+^ T cell population (**Figure 1G**). Although the percentages of T_FR_ cells in the whole population of pSS patients were similar to that of healthy controls, when patients were divided into two subgroups based on the presence or absence of autoantibodies, we found that the proportion of T_FR_ cells in patients having anti-Ro/SSA antibodies was significantly higher than those measured in autoantibody negative patients (*p* = 0.0363, **Figure 1G**). Regarding the absolute number of circulating T_FR_ cells, pSS patients had significantly decreased values compared to healthy individuals. This observation presumably stems from the fact that pSS patients have lower lymphocyte counts, which tends to bias the comparative analysis of absolute number of T cell subsets between pSS patients and healthy individuals (27, 28). Nevertheless, among patients, a significant elevation in absolute numbers of T_FR_ cell was detected in anti-Ro/SSA positive individuals, compared to autoantibody negative patients (*p* = 0.0157) (**Supplementary Figure 3F**).

### Altered Circulating T_FH_ Subset and T_FR_ Cell Frequencies Are Associated With Serological Findings

We investigated whether the alterations in activated cT_FH_ cells as well as T_FH_ cell subsets were associated with the disease etiology and other serological findings in pSS. Interestingly, the percentages of activated cT_FH_ cells showed a significant positive correlation with the levels of anti-La/SSB autoantibody (R = 0.3504; *p* = 0.0310) and with serum IgA titer (R = 0.3530; *p* = 0.0297) (**Figure 1H**). In addition, we found that the proportion of T_FH_1 (R = 0.3368; *p* = 0.0387) and T_FH_1/17 cells (R = 0.3734; *p* = 0.0209), but not of T_FH_2 cells, was significantly positively correlated with the levels of ICs (**Figure 1H**). The frequency of T_FH_1 cells also showed a significant positive correlation with the levels of serum IgG (R = 0.3977; *p* = 0.0134) and anti-La/SSB autoantibody (R = 0.3948; *p* = 0.0142) (**Figure 1H**). Interestingly, significant positive associations were found between the ratio of T_FR_ cells and levels of anti-La/SSB autoantibody (R = 0.5158, *p* = 0.0140; **Figure 1H**). Associations between the percentages of T_FR_ cells and other T_FH_ cell subsets are shown in the supplement, but it is worth mentioning that there was a positive correlation between the frequency of T_FR_ cells and activated T_FH_ cells (R = 0.5054, *p* = 0.0164) (**Supplementary Figure 4A**).

### Altered Distribution of Peripheral B Cell Subpopulations Is a Characteristic Feature of pSS

**Figure 2:**
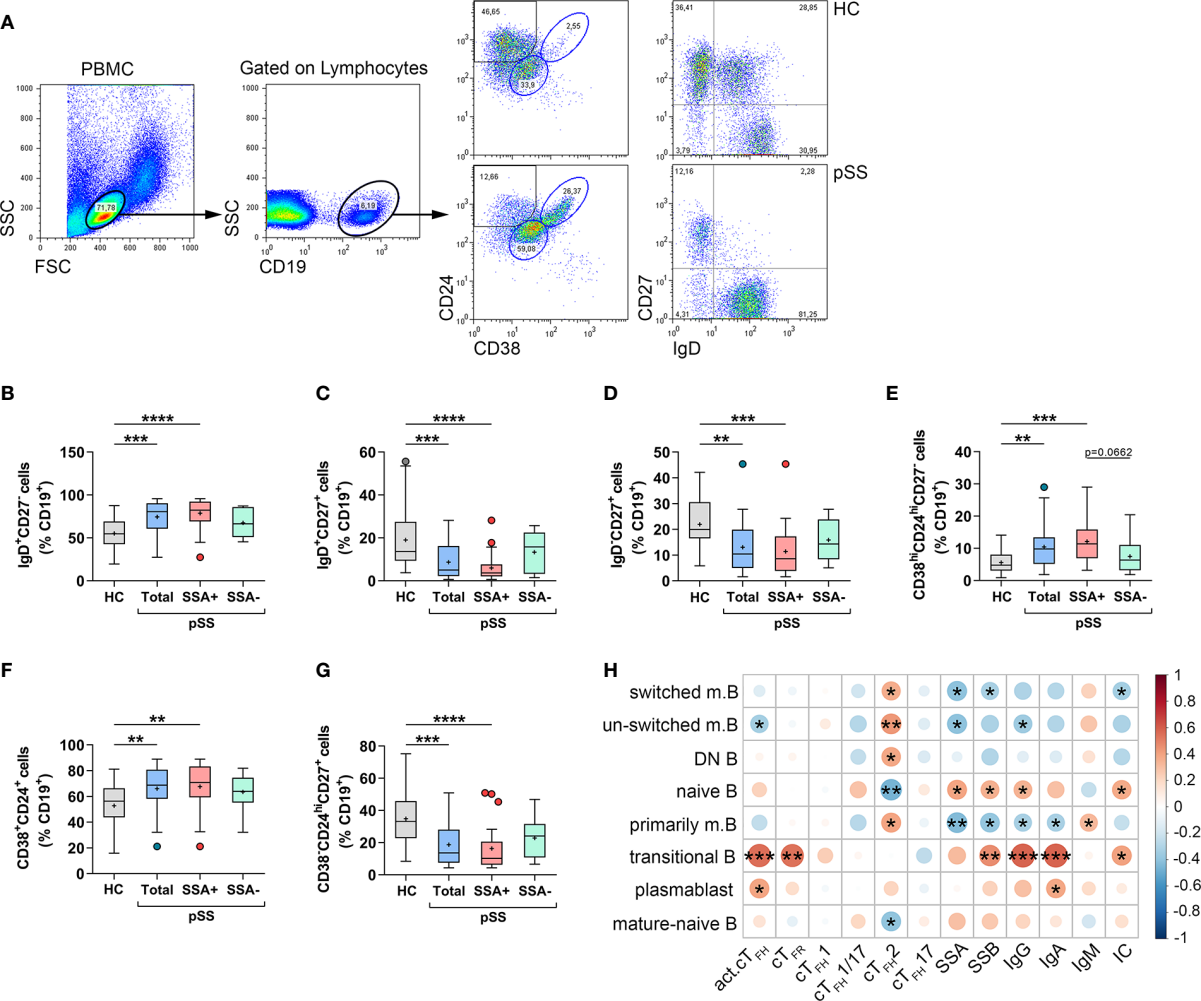
Abnormal distribution of different B cell subsets reflects the pathophysiology of pSS. Peripheral blood B cell subsets were quantified as their percentage in CD19^+^ lymphocyte population. **(A)** Representative dot plots indicate the gating strategy and determination of different B cell subsets. Percentages of IgD^+^CD27^-^ naive B cells **(B)**, IgD^+^CD27^+^ un-switched memory B cells **(C)**, IgD^-^CD27^+^ switched memory B cells **(D)**, CD38^hi^CD24^hi^CD27^−^ transitional B cells **(E)**, CD38^+^CD24^+^ mature-naive B cells **(F)**, CD38^-^CD24^hi^CD27^+^ primarily memory B cells **(G)** in pSS patients (n =38), pSS without anti-SSA(−) (n = 14) and pSS with anti-SSA(+) (n = 24) and healthy controls (HC; n = 29). **(H)** Frequencies of certain B cell subsets of pSS patients were acquired, and correlation analysis with serological parameters as well as with the percentages of cT_FH_ subsets was carried out. Data analyses were performed using one-way ANOVA with Tukey’s multiple comparisons test **(D, E)** or Kruskal–Wallis test with Dunn’s multiple comparisons test **(B, C, F, G)**. Correlation analysis was carried out with Spearman’s test **(H)**. Box plots represent the interquartile range (IQR) with a line in the middle as median and “+” sign as the mean value. Statistically significant differences are indicated by *p < 0.05; **p < 0.01; ***p < 0.001; ****p < 0.0001.

**Figure 3:**
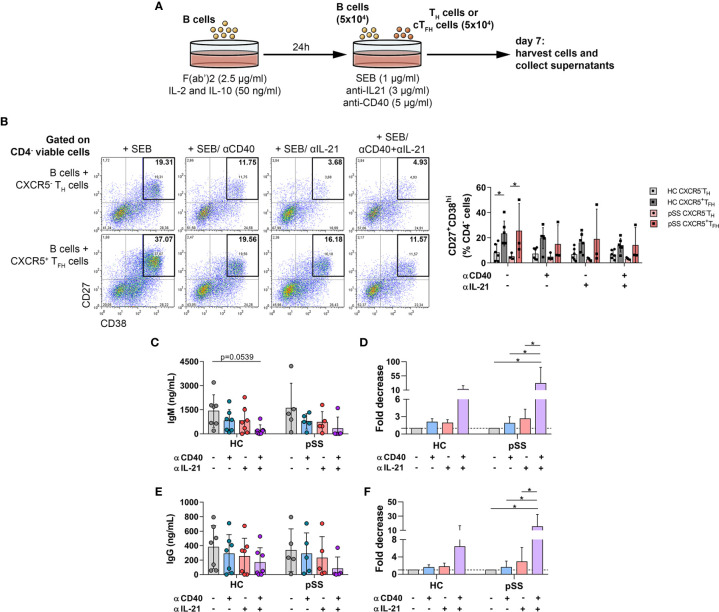
The effects of anti-human CD40/TNFRSF5 and anti-IL-21 on B-cell differentiation and antibody production. **(A)** Graphical representation of B-T_H_/cT_FH_ **** co-culture assay. Magnetically isolated CD19^+^ B cells were pre-activated with F(ab′)2 fragment in the presence of recombinant IL-2 and IL-10 for 24 h. Isolated ****CD4^+^CXCR5^+^ cT_FH_ or ****CD4^+^CXCR5^−^ T_H_ cells were co-cultured for 7 days with autologous B cells under stimulation by SEB and in the presence or absence of anti-human CD40****/TNFRSF5 and anti-human IL-21 antibodies. **(B)** Representative dot plots show the neutralization effect of anti-human CD40 and IL-21 on plasmablast differentiation. Percentages of 7-AAD^−^CD4^-^CD27^+^CD38^hi^ viable plasmablasts were measured by flow cytometry in healthy controls (HC; n = 6) and pSS patients (n = 3). Two-way ANOVA followed by Bonferroni’s posttest was used. Bars represent mean with SD. **(C–F)** Concentration and fold decrease of IgM and IgG were measured by ELISA in patients with pSS (n = 5) and HCs (n = 7). Bars show the mean with SD and each data point represents an individual subject. Data analysis performed with two-way ANOVA followed by Tukey’s multiple comparison tests. Statistically significant differences are defined as *p < 0.05.

In parallel with the assessment of circulating T_FH_ subsets in pSS, we measured the proportions of different B cell subpopulations, in order to get a better view on T_FH_–B cell axis in pSS pathogenesis. Lymphocytes were first gated on their characteristic scatter pattern and then gated on the CD19^+^ population. Memory B cell subsets and naïve or mature-naïve B cells were identified according to IgD, CD27, CD38, and CD24 expression (**Figure 2A**). Proportions of CD19^+^IgD^+^CD27^−^ naive B cells were significantly increased in pSS patients compared to healthy individuals (*p* = 0.0009, **Figure 2B**). As expected, patients with anti-Ro/SSA seropositivity had significantly higher percentages than controls (*p* < 0.0001, **Figure 2B**). On the other hand, the frequency of CD19^+^IgD^+^CD27^+^ un-switched memory B cells was significantly diminished in the total pSS population (*p* = 0.0010, **Figure 2C**), while the difference was more pronounced in patients with anti-Ro/SSA antibodies (*p* < 0.0001, **Figure 2C**) compared to controls. Interestingly, the proportion of un-switched memory B cells in autoantibody-negative patients was similar to control results (**Figure 2C**). Peripheral CD19^+^IgD^−^CD27^+^ switched memory B-cell percentages closely followed the above described tendency, since they were significantly reduced in the whole pSS patient group compared to controls (*p* = 0.0016, **Figure 2D**). Moreover, the anti-Ro/SSA positive group showed a significant diminished ratio compared to healthy controls (*p* = 0.0007, **Figure 2D**), as well. The percentages of CD19^+^CD38^hi^CD24^hi^CD27^−^ transitional B cells were significantly elevated in the total patient group (*p* = 0.0029), along with anti-Ro/SSA antibody positive patients (*p* = 0.0002) (**Figure 3E**) compared to control values. On the other hand, we found that the proportion of transitional B cells had a tendency to be increased in patients having autoantibodies compared to the autoantibody-negative patient population (*p* = 0.0662, **Figure 2E**). The ratio of CD19^+^CD38^int^CD24^int^ mature-naïve B-cell subset followed a similar tendency as naïve B cells (HC *vs.* Total: *p* = 0.0064; HC *vs.* aSSA+: *p* = 0.0038, **Figure 2F**). The percentages of CD19^+^CD38^−^CD24^hi^CD27^+^ primarily memory B cells were significantly diminished in the total pSS patients (*p* = 0.0005) as well as in the anti-Ro/SSA subgroup (*p* < 0.0001) (**Figure 2G**) in comparison with healthy subjects.

Upon assessing the associations between B cell subpopulations and other measured parameters, we identified a significant negative correlation between the levels of serum anti-Ro/SSA and the frequency of switched memory B (R = −0.4047; *p* = 0.0117), un-switched memory B (R = −0.4046; *p* = 0.0118) and primarily memory B cells (R = −0.4311; *p* = 0.0069), while they showed positive correlation with naïve B cell percentages (R = 0.3957; *p* = 0.0139) (**Figure 2H**). The presence of serum anti-La/SSB showed a significant negative correlation with switched memory B (R = −0.3232; *p* = 0.0478) and primarily memory B cells (R = −0.3888; *p* = 0.0159), whereas their serum concentrations revealed a significant positive association with the frequencies of naïve B (R = 0.3532; *p* = 0.0296) as well as transitional B cells (R = 0.4641; *p* = 0.0033) (**Figure 2H**). The levels of IgG and IgA showed a similar correlation as autoantibodies, since they correlated negatively with the proportion of primarily memory B cells (IgG: R = −0.3258; *p* = 0.0459, IgA: R = −0.3233; *p* = 0.0477), while IgG also displayed a significant positive association with the percentages of naïve B cells (R = 0.3490; *p* = 0.0318) and a significant negative correlation with the frequency of un-switched memory B (R = −0.3570; *p* = 0.0278) (**Figure 2H**). Interestingly, we found a positive association between the percentages of transitional B cells and serum levels of IgG (R = 0.5861; *p* = 0.0001) and IgA (R = 0.5882; *p* = 0.0001) (**Figure 2H**). Levels of serum IgA also showed a significant positive correlation with the percentages of plasmablasts (R = 0.3821; *p* = 0.0179) while IgM exhibited a positive correlation with primarily memory B cells (R = 0.3237; *p* = 0.0474) (**Figure 2H**). Moreover, the presence of serum ICs correlated positively with naive (R = 0.3794; *p* = 0.0188) and transitional B cells (R = 0.4049; *p* = 0.0117), and negatively with switched memory B cells (R = −0.3302; *p* = 0.0429) (**Figure 2H**).

We also assessed the possible associations between peripheral T_FH_ cell subsets and B cell subpopulations. The frequency of activated cT_FH_ cells within CD4^+^CXCR5^+^ cells revealed a significant positive correlation with the percentages of transitional B cells (R = 0.5520; *p* = 0.0003) and plasmablasts (R = 0.3960; *p* = 0.0139), whereas it showed a significant negative correlation with un-switched memory B cell proportions (R = −0.3345; *p* = 0.0401) (**Figure 2H**). Interestingly, only the frequency of T_FH_2 subset showed any association with B cell subsets. A significant positive correlation was found with every memory B cell subpopulation (switched B: R = 0.3640; *p* = 0.0246, un-switched B: R = 0.4301; *p* = 0.0070, DN B: R = 0.3826; *p* = 0.0177; primarily memory B: R = 0.3986; *p* = 0.0132), while a significant negative correlation was observed with the frequency of naïve B cells (R = −0.4645; *p* = 0.0033) and mature-naïve B cells (R = −0.3967; *p* = 0.0137) (**Figure 2H**). Of note, significant positive associations were found between the proportion of T_FR_ cells and transitional B cells (R = 0.5438, *p* = 0.0089) (**Figure 2H**). In summary, the present results reinforced our previously published observations on the distribution of B cell subsets in pSS (29). Moreover, our current findings on the proportions of certain B cell subsets along with the high percentages of activated cT_FH_ cells and T_FR_ cells, point at the crucial role of T_FR_–T_FH_–B cell axis in the development of aberrant humoral immunity in pSS.

### T_FH_ Cells Help B Cells in the Peripheral Blood of pSS Patients

It is known that T_FH_ cells play an important role in B-cell proliferation and high-affinity antibody production by elevated expression of costimulatory signals as well as secreting stimulating cytokines, including IL-21. Our aforementioned results suggested a direct association between activated T_FH_ cells and B cells in the early differentiation state or with antibody production. To further investigate this issue, we co-cultured B cells with isolated T helper or circulating T_FH_ cells *in vitro* in the presence of SEB superantigen and investigated the competence of certain T cell subsets to induce B cell differentiation into CD27^+^CD38^hi^ plasmablasts (**Figure 3A**). In order to reinforce the key function of T_FH_ cells in pSS, we investigated the neutralization of T and B-cell interaction in co-culture, blocking antibodies against IL-21, and CD40/TNFRSF5 was added in T–B co-cultures. As expected, cT_FH_ cells supported B-cell differentiation and plasmablast development more efficiently than CD4^+^CXCR5^−^ T cells in both controls and pSS patients (HC untreated: *p* = 0.0314 and pSS untreated: *p* = 0.0432, **Figure 3B**), but we failed to detect any difference after neutralization. The results of pSS patients and controls in IgM and IgG production showed similar patterns, but a tendency towards lower production of IgM in healthy individuals after simultaneous treatment with anti-IL-21 and anti-CD40 was evident (*p* = 0.0539, **Figure 3C**). When we measured the fold decrease in the production of IgG and IgM antibodies, patients with pSS appeared to be more affected by both neutralizations than healthy individuals. There was a 31-fold decrease in IgM secretion by the treatment with both CD40 and IL-21 blockade in patients with pSS (*p* = 0.0125, **Figure 3D**) compared to a 12-fold decrease in healthy subjects. In case of IgG production, we found a 15-fold decrease after anti-CD40 and anti-IL-21 treatment in pSS patients (*p* = 0.0105, **Figure 3F**) in comparison with a six-fold decrease in controls. These data reinforce the importance of the T_FH_–B cell axis in the development of aberrant humoral immunity in pSS.

## Discussion

Disturbance of B-cell homeostasis and autoantibody production are key hallmarks of pSS (30). We have previously shown the important role of T_FH_ cells and their IL-21 secretion in autoreactive B cell activation and autoantibody production in pSS (26). Recently, we also demonstrated decreased percentages of peripheral IL-10^+^ regulatory B cells in pSS and confirmed the strong relationship between IL-21 production of blood T_FH_ cells and the characteristic aberrant distribution of circulating B-cell subsets in the disease (29). In the present study, we confirmed the increased frequency of activated cT_FH_ cells in an independent cohort of pSS patients and that their altered distribution is associated with anti-Ro/SSA seropositivity. Previous studies have examined circulating ICOS^+^PD-1^+^ T_FH_ cells in pSS patients and have found a correlation with ESSDAI or with the presence of ectopic lymphoid structures (31–33). Other studies focused on CCR7^lo^PD-1^+^ or PD-1^+^ cT_FH_ cells and found a positive association with the degree of focal lymphocytic infiltration in the salivary gland and also with ESSDAI (34, 35). An important difference between our study and former research is that we included only patients with low disease activity without the necessity of ongoing immunosuppressive treatment. We investigated the distribution of recently described subtypes of cT_FH_ cells in a relatively large number of pSS patients. Although the ratio of these cells did not differ significantly in pSS patients compared to healthy controls, the percentages of T_FH_1 cells tended to increase within the CD4^+^CXCR5^+^ cT_FH_ pool. However, the alteration in the distribution of T_FH_ subsets is still a matter of debate. The previously published data of Li et al. described that frequencies of Th17-like cells within CXCR5^+^CD4^+^ T cells were significantly higher in pSS patients than in healthy controls (36). In contrast, another study showed that the frequency of circulating CXCR5^+^ Th17 cells is decreased in pSS patients, as a result of their extensive infiltration towards the site of the inflammation related to the local expression of CCL20 ligand (37). In support of our data, Kim et al. recently reported similar distributions of T_FH_ cell subsets in pSS compared to healthy subjects (34), Interestingly, patients with IgG4-related disease (RD) are also in the center of interest concerning T_FH_ research, but the results are more in agreement with the original work by Morita et al. (15) stating that CXCR5^+^ Th2 and Th17 cells are more proficient B cell helpers than the Th1 phenotype. In line with this, Akiyama et al. described that only cT_FH_2 cells induce the differentiation of naïve B cells, enhance the production of IgG4, and associate with disease activity in patients with untreated IgG4-RD (38). Based on the similarity of organ involvement, pSS patients were enrolled as controls in that study, but did not show any difference in T_FH_ subsets compared to healthy individuals (38). In another investigation, IgG4-RD was also characterized by a shift toward Th2/T_FH_2 and Th17/T_FH_17 polarization, but patients with pSS failed to show any abnormality in the T_FH_ subsets (39). Our data are in support of no specific abnormality within T_FH_ subsets, but rather an enhanced activation status.

Since their discovery, cT_FH_ cell subsets were investigated extensively in several autoimmune diseases, including systemic lupus erythematosus (40–44), rheumatoid arthritis (45–48), myasthenia gravis (49), multiple sclerosis (50), and IgA vasculitis (51) with varying results. Considering the contradictions as well as limited amount of data in pSS, we attempted to resolve this discrepancy and clarify the distribution of blood T_FH_ subpopulations. Since the assessment of peripheral B cells is lacking in several previous studies (33, 52), we measured a wide spectrum of B-cell subsets to assess how their characteristic distribution correlates with the proportions of T_FH_ subsets.

We correlated the expression of activation markers ICOS and PD-1 with the percentages of cT_FH_ subsets and discovered that mainly cT_FH_1 cells were activated while cT_FH_17 and cT_FH_2 cells were probably in a quiescent state in the peripheral blood of patients with pSS. Correlation analysis with serological markers reinforced our previous findings that activated cT_FH_ cells are in relation with serum IgA and anti-La/SSB but regarding the cell subsets, only cT_FH_1 and partially cT_FH_1/17 cells seem to play a role in the pathogenesis of pSS. In order to correlate the ratio of cT_FH_ cells and B cells, we measured a wide variety of B cell subpopulations. Our results on B cells confirmed previous observations (29); moreover, the present pSS patient group showed a more characteristic imbalance regarding memory B cells and transitional B-cell distributions. Hence, according to our data, significant correlation with B cells was restricted only to activated cT_FH_ and cT_FH_2 cells. Activated cT_FH_ showed negative correlation with un-switched memory B cells and correlated positively with transitional B cells and plasmablasts, while, cT_FH_2 cells correlated positively with memory B cell subtypes and demonstrated negative association with naïve and mature-naive B cells. Inevitably, these data raise the question of how the counterintuitive decrease in memory B cell and altered cT_FH_ cell frequencies could contribute to humoral autoimmunity in pSS. Central and peripheral tolerance checkpoints are known to be defective in patients with autoimmune diseases, leading to the expansion of transitional and naïve autoreactive B cells in peripheral blood. A recent study has demonstrated that activated naive B cells could generate highly polyclonal antibody-secreting cells and could be responsible for serum autoantibody repertoire in lupus (53). From several studies it has become clear that the decreased ratio of peripheral memory B cells may be interpreted by the upregulation of CXCR4 and CXCR3 chemokines which arrange their recruitment into the inflamed tissues of affected organs (3, 54–57). Ectopic expression of chemokines, including CXCL13, CCL21, and CXCL12 not only induces the migration and survival of memory B cells and long-lived plasma cells, but supports lymphoid neogenesis in the pSS salivary glands (58). Upregulated expression of CXCL10 and CCL20 in the salivary gland tissue of pSS raises the possibility that an altered T_FH_ subset distribution at the periphery may be a consequence of their migration towards the affected organs (37).

Besides revealing the alterations and associations of the distribution of cT_FH_ cell subsets, the present study also explored the possible importance of T_FR_ cells in pSS. Our study defined peripheral T_FR_ cells as CD127^lo/−^CD25^hi^ within CD4^+^CXCR5^+^ cT_FH_ cells and found that their frequency and absolute number were increased in pSS patients with seropositivity for anti-Ro/SSA autoantibody compared to seronegative patients. In addition, we investigated the relationship between T_FR_ cells and the investigated T_FH_ subsets and demonstrated a positive correlation with activated cT_FH_ and cT_FH_1 cells, whereas a negative correlation was found with cT_FH_17 cells. Furthermore, the ratio of T_FR_ cells also showed a positive association with the percentages of transitional B cells as well as the serum levels of anti-La/SSB and IgM. Our observations on T_FR_ ratios are in accordance with the recent findings of Kim et al. and Fonseca et al., who also reported such increased percentages of T_FR_ cells in pSS (34, 59). Additionally, when the latter workgroup compared the suppressive capacity of T_FR_ cells and CXCR5^−^ Treg cells, they found that T_FR_ cells do not have specialized humoral regulatory capacity, but these cells were able to up-regulate CXCR5 upon *in vitro* activation and migrate towards CXCL13 gradient, therefore circulating T_FR_ cells could be recruited into subsequent GC responses (59). It is assumed, that blood T_FR_ cells form before the full differentiation into follicle resident T_FR_ cells and retain the expression of CD25 and CXCR5, but lack BCL6 expression as well as the humoral suppression capacity of the mature phenotype (60). Therefore, the increase in cT_FR_ ratio in the peripheral blood could indicate an ongoing humoral response and not a measure of suppressor activity (31, 59). Possibly, the elevated IL-21 production during the follicular and GC phase inhibits T_FR_ commitment through the down-regulation of Foxp3 and CD25 (61, 62), thus the elevation of pre-T_FR_ cells and their traffic to the periphery could be a part of an increased counter-regulatory reaction. Hence, the negative correlation of T_FR_/T_FH_17 in the peripheral blood may be not the outcome of a T_FR_-mediated suppression, but it is rather derived from the imbalance of the differentiation process in secondary lymphoid tissue.

Costimulatory and cytokine-mediated signals are needed to support T cell-dependent B-cell responses (8). Since CD40 ligation is necessary for the survival and differentiation of GC B cells, blockade of CD40L signaling or disruption of CD40L–CD40 interaction could be beneficial for preventing autoantibody production and B cell abnormality (63). The role of this interaction has been implicated by others, as an increased expression of CD40 has been observed in the salivary gland of pSS and in mice (64, 65). IL-21 is a pleiotropic cytokine, mainly secreted by T_FH_ cells, and it signals through IL-21 receptor which is broadly expressed by different types of lymphoid and myeloid cells. This cytokine has a crucial role not only in the development of T_FH_ cells but also in the differentiation of B cells and the generation of humoral immune responses as well (66). Of note, *in vitro* analyses on T_FH_ mediated B cell responses with blocking CD40L–CD40 interaction and neutralizing IL-21 effects in pSS were previously performed only in animal models (67, 68). To further clarify the role of costimulation in our *in vitro* assay, anti-human CD40/TNFRSF5 and anti-human IL-21 antibodies were added together or by itself to inhibit T_FH_-B-cell interaction. To examine their functions, CXCR5^−^ T helper and CXCR5^+^ cT_FH_ cells were isolated from CD4^+^ lymphocytes. As expected, blood T_FH_ cells offered more efficient B-cell help than conventional T helper cells, resulting in the generation of more CD19^lo^CD27^+^CD38^hi^ plasmablasts in the T–B co-culture in both patients and controls. Nevertheless, B cells co-cultured with CD4^+^CXCR5^−^ T cells were still able to differentiate into plasmablast. Of note, a recent study defined a population of PD-1^hi^ expressing CD4^+^CXCR5^−^ peripheral helper T (T_PH_) cells that effectively induce the formation of ectopic lymphoid structures by recruiting B cells *via* CXCL13 secretion and support their survival and maturation by the production of IL-21 (69). There was a clear difference in the expression of factors associated with B-cell help between the PD-1^−^ and PD-1^hi^ populations within the CD4^+^CXCR5^−^ T cells for the benefit of the latter; presumably this subset interfered with our results. Blockade of CD40 decreased the production of both IgM and IgG in the *in vitro* assay, suggesting a CD40L–CD40 dependent interaction between CD4^+^ T cells and B cells. Neutralization of human IL-21 bioactivity affected IgG and IgM production similarly, but simultaneous treatment with both substances proved to be more effective. When we analyzed the fold-decrease of Ig production, we were able to demonstrate a difference between patients with pSS and healthy individuals, since the neutralization effect was more pronounced in pSS. The limitation of our study is the use of total CD4^+^CXCR5^+^ T and CD19^+^ B cells in our functional analysis, as the B-cell population consists of an increased proportion of naive B cells in patients with pSS; furthermore cT_FR_ cells probably occur within CD4^+^CXCR5^+^ T cells. Since Fonseca et al. demonstrated in a functional assay that blood T_FR_ cells suppress T_FH_ cell and naïve B cell proliferation, thus it could have influenced the outcome of the assay in pSS patients compared to controls (59). Other limitations are the inclusion of patients with a broad range of disease duration and small sample size in the functional analysis.

Of note, the blockade of the CD40–CD40L co-stimulatory pathway is a promising therapeutic approach in pSS. A phase II study on subcutaneous or intravenous administration of an inhibitory monoclonal antibody against CD40 (iscalimab/CFZ533) revealed a decrease in circulating CXCL13 levels and B-cell activation (70). Another study demonstrated that there was no significant difference in ESSDAI score between subcutaneous iscalimab and placebo; therefore therapeutic potential for CD40 blockade in pSS should be further investigated (71).

In conclusion, our findings suggest that there may be no detectable imbalance in the distribution of cT_FH_ subsets in the periphery of pSS. However, our study points out that increased ratios of activated cT_FH_ cells and T_FR_ cells are involved in the pathogenesis of pSS by providing help to B cells. Neutralization of CD40 and/or IL-21 reduced antibody production more effectively in pSS than in healthy controls *in vitro*; thus, the interruption of T and B-cell collaboration may be a potential therapeutic approach for down-regulating pathological humoral immunity in pSS.

## Data Availability Statement

The original contributions presented in the study are included in the article/**Supplementary Material**. Further inquiries can be directed to the corresponding author.

## Ethics Statement

The studies involving human participants were reviewed and approved by the Ethics Committee of our University (protocol number: 4879-2017) and the Policy Administration Services of Public Health of the Government Office (protocol number: 1660-4/2018). All data stored and all experiments carried out were in compliance with the Declaration of Helsinki. The patients/participants provided their written informed consent to participate in this study.

## Author Contributions

KS designed and performed the experiments, analyzed data, prepared the figures, and wrote the main manuscript text. IJ performed the experiments. AS and IH directed the clinical study and collected human samples. TT, BN, and PS provided conceptual advices and edited the manuscript. GP designed and financed the study and contributed to the final version of the manuscript. All authors contributed to the article and approved the submitted version.

## Funding

This work was supported by the National Research, Development and Innovation Office (NKFIH K 124177 to GP, PD 121327 to KS) (Hungary).

## Conflict of Interest

The authors declare that the research was conducted in the absence of any commercial or financial relationships that could be construed as a potential conflict of interest.

## References

[1] Brito-Zerón P Baldini C Bootsma H Bowman S Jonsson R Mariette X. Sjögren syndrome. Nat Rev Dis Primers (2016) 2:16047. 10.1038/nrdp.2016.47 27383445

[2] Ittah M Miceli-Richard C Eric Gottenberg J Lavie F Lazure T Ba N. B cell-activating factor of the tumor necrosis factor family (BAFF) is expressed under stimulation by interferon in salivary gland epithelial cells in primary Sjögren’s syndrome. Arthritis Res Ther (2006) 8(2):R51. 10.1186/ar1912 16507175 PMC1526588

[3] Rivière E Pascaud J Tchitchek N Boudaoud S Paoletti A Ly B. Salivary gland epithelial cells from patients with Sjögren’s syndrome induce B-lymphocyte survival and activation. Ann Rheum Dis (2020) 79(11):1468–77. 10.1136/annrheumdis-2019-216588 32843324

[4] Mackay F Groom J Tangye S. An important role for B-cell activation factor and B cells in the pathogenesis of Sjögren’s syndrome. Curr Opin Rheumatol (2007) 19(5):406–13. 10.1097/BOR.0b013e328277ef4c 17762603

[5] Corsiero E Nerviani A Bombardieri M Pitzalis C. Ectopic Lymphoid Structures: Powerhouse of Autoimmunity. Front Immunol (2016) 7:430. 10.3389/fimmu.2016.00430 27799933 PMC5066320

[6] Kim C Rott L Clark-Lewis I Campbell D Wu L Butcher E. Subspecialization of CXCR5+ T cells: B helper activity is focused in a germinal center-localized subset of CXCR5+ T cells. J Exp Med (2001) 193(12):1373–81. 10.1084/jem.193.12.1373 PMC2193300 11413192

[7] Horiuchi S Ueno H. Potential Pathways Associated With Exaggerated T Follicular Helper Response in Human Autoimmune Diseases. Front Immunol (2018) 9:1630:1630. 10.3389/fimmu.2018.01630 30061896 PMC6054970

[8] Vinuesa CG Linterman MA Yu D MacLennan IC. Follicular Helper T Cells. Annu Rev Immunol (2016) 34:335–68. 10.1146/annurev-immunol-041015-055605 26907215

[9] Qi H. T follicular helper cells in space-time. Nat Rev Immunol (2016) 16(10):612–25. 10.1038/nri.2016.94 27573485

[10] Chevrier S Kratina T Emslie D Tarlinton D Corcoran L. IL4 and IL21 Cooperate to Induce the High Bcl6 Protein Level Required for Germinal Center Formation. Immunol Cell Biol (2017) 95(10):925–32. 10.1038/icb.2017.71 28875978

[11] DeFranco A. Germinal centers and autoimmune disease in humans and mice. Immunol Cell Biol (2016) 94(10):918–24. 10.1038/icb.2016.78 PMC5663225 27562062

[12] Ueno H. Human Circulating T Follicular Helper Cell Subsets in Health and Disease. J Clin Immunol (2016) 36 Suppl 1:34–9. 10.1007/s10875-016-0268-3 26984851

[13] Schmitt N Bentebibel SE Ueno H. Phenotype and functions of memory Tfh cells in human blood. Trends Immunol (2014) 35(9):436–42. 10.1016/j.it.2014.06.002 PMC4152409 24998903

[14] He J Tsai L Leong Y Hu X Ma C Chevalier N. Circulating precursor CCR7(lo)PD-1(hi) CXCR5⁺ CD4⁺ T cells indicate Tfh cell activity and promote antibody responses upon antigen reexposure. Immunity (2013) 39(4):770–81. 10.1016/j.immuni.2013.09.007 24138884

[15] Morita R Schmitt N Bentebibel S Ranganathan R Bourdery L Zurawski G. Human blood CXCR5(+)CD4(+) T cells are counterparts of T follicular cells and contain specific subsets that differentially support antibody secretion. Immunity (2011) 34(1):108–21. 10.1016/j.immuni.2010.12.012 PMC3046815 21215658

[16] Locci M Havenar-Daughton C Landais E Wu J Kroenke M Arlehamn C. Human circulating PD-1+CXCR3-CXCR5+ memory Tfh cells are highly functional and correlate with broadly neutralizing HIV antibody responses. Immunity (2013) 39(4):758–69. 10.1016/j.immuni.2013.08.031 PMC3996844 24035365

[17] Boswell K Paris R Boritz E Ambrozak D Yamamoto T Darko S. Loss of circulating CD4 T cells with B cell helper function during chronic HIV infection. PloS Pathog (2014) 10(1):e1003853. 10.1371/journal.ppat.1003853 24497824 PMC3911819

[18] Bentebibel S Lopez S Obermoser G Schmitt N Mueller C Harrod C. Induction of ICOS+CXCR3+CXCR5+ TH cells correlates with antibody responses to influenza vaccination. Sci Transl Med (2013) 5(176):176ra32. 10.1126/scitranslmed.3005191 PMC3621097 23486778

[19] Fonseca V Ribeiro F Graca L. T follicular regulatory (Tfr) cells: Dissecting the complexity of Tfr-cell compartments. Immunol Rev (2019) 288(1):112–27. 10.1111/imr.12739 30874344

[20] Vitali C Bombardieri S Jonsson R Moutsopoulos H Alexander E Carsons S. Classification criteria for Sjögren’s syndrome: a revised version of the European criteria proposed by the American-European Consensus Group. Ann Rheum Dis (2002) 61:554–8. 10.1136/ard.61.6.554 PMC1754137 12006334

[21] Shiboski CH Shiboski SC Seror R Criswell LA Labetoulle M Lietman TM. 2016 American College of Rheumatology/European League Against Rheumatism classification criteria for primary Sjögren’s syndrome: A consensus and data-driven methodology involving three international patient cohorts. Ann Rheum Dis (2017) 76(1):9–16. 10.1136/annrheumdis-2016-210571 27789466

[22] Seror R Ravaud P Bowman SJ Baron G Tzioufas A Theander E. EULAR Sjogren’s syndrome disease activity index: development of a consensus systemic disease activity index for primary Sjogren’s syndrome. Ann Rheum Dis (2010) 69(6):1103–9. 10.1136/ard.2009.110619 PMC2937022 19561361

[23] R Development Core Team. A language and environment for statistical computing. 3.4.0 ed. Vienna, Austria: R Foundation for Statistical Computing; 2017.

[24] Wie T Simko V. R package “corrplot”: Visualization of a Correlation Matrix (Version 0.84). (2017). Available at: https://githubcom/taiyun/corrplot. [Accessed March 12, 2021].

[25] Harrell FJnt. Hmisc: Harrell Miscellaneous. R package version 4.4-0. (2020). Available at: https://CRANR-projectorg/package=Hmisc. [Accessed March 12, 2021].

[26] Szabo K Papp G Barath S Gyimesi E Szanto A Zeher M. Follicular helper T cells may play an important role in the severity of primary Sjögren’s syndrome. Clin Immunol (2013) 147(2):95–104. 10.1016/j.clim.2013.02.024 23578551

[27] Schulze-Koops H. Lymphopenia and autoimmune diseases. Arthritis Res Ther (2004) 6(4):178–80. 10.1186/ar1208 PMC464928 15225363

[28] Mandl T Bredberg A Jacobsson LT Manthorpe R Henriksson G. CD4+ T-lymphocytopenia–a frequent finding in anti-SSA antibody seropositive patients with primary Sjögren’s syndrome. J Rheumatol (2004) 31(4):726–8. 15088298

[29] Szabó K Papp G Szántó A Tarr T Zeher M. A comprehensive investigation on the distribution of circulating follicular T helper cells and B cell subsets in primary Sjögren’s syndrome and systemic lupus erythematosus. Clin Exp Immunol (2016) 183(1):76–89. 10.1111/cei.12703 26358223 PMC4687513

[30] Garcillán B Figgett W Infantino S Lim E Mackay F. Molecular control of B-cell homeostasis in health and malignancy. Immunol Cell Biol (2018) 96(5):453–62. 10.1111/imcb.12030 29499091

[31] Fonseca V Romão V Agua-Doce A Santos M López-Presa D Ferreira A. The Ratio of Blood T Follicular Regulatory Cells to T Follicular Helper Cells Marks Ectopic Lymphoid Structure Formation While Activated Follicular Helper T Cells Indicate Disease Activity in Primary Sjögren’s Syndrome. Arthritis Rheumatol (2018) 70(5):774–84. 10.1002/art.40424 29361207

[32] Verstappen GM Nakshbandi U Mossel E Haacke EA van der Vegt B Vissink A. Is the T Follicular Regulatory:Follicular Helper T Cell Ratio in Blood a Biomarker for Ectopic Lymphoid Structure Formation in Sjögren’s Syndrome? Comment on the Article by Fonseca et al. Arthritis Rheumatol (2018) 70(8):1354–5. 10.1002/art.40488 29534333

[33] Pontarini E Murray-Brown WJ Croia C Lucchesi D Conway J Rivellese F. Unique expansion of IL-21+ Tfh and Tph cells under control of ICOS identifies Sjögren’s syndrome with ectopic germinal centres and MALT lymphoma. Ann Rheum Dis (2020) 79(12):1588–99. 10.1136/annrheumdis-2020-217646 PMC7677495 32963045

[34] Kim J Lee J Hong S Lee J Cho M Park S. Circulating CCR7loPD-1hi Follicular Helper T Cells Indicate Disease Activity and Glandular Inflammation in Patients with Primary Sjögren’s Syndrome. Immune Netw (2019) 19(4):e26. 10.4110/in.2019.19.e26 31501714 PMC6722269

[35] Liu R Su D Zhou M Feng X Li X Sun L. Umbilical cord mesenchymal stem cells inhibit the differentiation of circulating T follicular helper cells in patients with primary Sjögren’s syndrome through the secretion of indoleamine 2,3-dioxygenase. Rheumatol (Oxford) (2015) 54(2):332–42. 10.1093/rheumatology/keu316 25169988

[36] Li X Wu Z Ding J Zheng Z Li X Chen L. Role of the frequency of blood CD4(+) CXCR5(+) CCR6(+) T cells in autoimmunity in patients with Sjögren’s syndrome. Biochem Biophys Res Commun (2012) 422(2):238–44. 10.1016/j.bbrc.2012.04.133 22575453

[37] Blokland SLM Kislat A Homey B Smithson GM Kruize AA Radstake TRDJ. Decreased circulating CXCR3 + CCR9+T helper cells are associated with elevated levels of their ligands CXCL10 and CCL25 in the salivary gland of patients with Sjögren’s syndrome to facilitate their concerted migration. Scand J Immunol (2020) 91(3):e12852. 10.1111/sji.12852 31733111 PMC7064901

[38] Akiyama M Yasuoka H Yamaoka K Suzuki K Kaneko Y Kondo H. Enhanced IgG4 production by follicular helper 2 T cells and the involvement of follicular helper 1 T cells in the pathogenesis of IgG4-related disease. Arthritis Res Ther (2016) 18:167. 10.1186/s13075-016-1064-4 27411315 PMC4944254

[39] Grados A Ebbo M Piperoglou C Groh M Regent A Samson M. T Cell Polarization toward TH2/TFH2 and TH17/TFH17 in Patients with IgG4-Related Disease. Front Immunol (2017) 8:235. 10.3389/fimmu.2017.00235 28348556 PMC5347096

[40] Le Coz C Joublin A Pasquali J Korganow A Dumortier H Monneaux F. Circulating TFH subset distribution is strongly affected in lupus patients with an active disease. PloS One (2013) 8(9):e75319. 10.1371/journal.pone.0075319 24069401 PMC3777901

[41] Choi J Ho J Pasoto S Bunin V Kim S Carrasco S. Circulating follicular helper-like T cells in systemic lupus erythematosus: association with disease activity. Arthritis Rheumatol (2015) 67(4):988–99. 10.1002/art.39020 PMC4450082 25581113

[42] Xu H Liu J Cui X Zuo Y Zhang Z Li Y. Increased frequency of circulating follicular helper T cells in lupus patients is associated with autoantibody production in a CD40L-dependent manner. Cell Immunol (2015) 295(1):46–51. 10.1016/j.cellimm.2015.01.014 25748125

[43] Xu B Wang S Zhou M Huang Y Fu R Guo C. The ratio of circulating follicular T helper cell to follicular T regulatory cell is correlated with disease activity in systemic lupus erythematosus. Clin Immunol (2017) 183:46–53. 10.1016/j.clim.2017.07.004 28709914 PMC5673570

[44] Liu C Wang D Song Y Lu S Zhao J Wang H. Increased circulating CD4+CXCR5+FoxP3+ follicular regulatory T cells correlated with severity of systemic lupus erythematosus patients. Int Immunopharmacol (2018) 56:261–8. 10.1016/j.intimp.2018.01.038 29414660

[45] Arroyo-Villa I Bautista-Caro M Balsa A Aguado-Acín P Bonilla-Hernán M Plasencia C. Constitutively altered frequencies of circulating follicullar helper T cell counterparts and their subsets in rheumatoid arthritis. Arthritis Res Ther (2014) 16(6):500. 10.1186/s13075-014-0500-6 25475240 PMC4275955

[46] Singh D Henkel M Sendon B Feng J Fabio A Metes D. Analysis of CXCR5+Th17 cells in relation to disease activity and TNF inhibitor therapy in Rheumatoid Arthritis. Sci Rep (2016) 6:39474. 10.1038/srep39474 28004828 PMC5177940

[47] Costantino A Acosta C Onetti L Mussano E Cadile I Ferrero P. Follicular helper T cells in peripheral blood of patients with rheumatoid arthritis. Reumatol Clin (2017) 13(6):338–43. 10.1016/j.reuma.2016.07.003 27595364

[48] Niu Q Huang Z Wu X Jin Y An Y Li Y. Enhanced IL-6/phosphorylated STAT3 signaling is related to the imbalance of circulating T follicular helper/T follicular regulatory cells in patients with rheumatoid arthritis. Arthritis Res Ther (2018) 20(1):200. 10.1186/s13075-018-1690-0 30157931 PMC6116385

[49] Zhang CJ Gong Y Zhu W Qi Y Yang CS Fu Y. Augmentation of Circulating Follicular Helper T Cells and Their Impact on Autoreactive B Cells in Myasthenia Gravis. J Immunol (2016) 197(7):2610–7. 10.4049/jimmunol.1500725 27543617

[50] Romme Christensen J Börnsen L Ratzer R Piehl F Khademi M Olsson T. Systemic inflammation in progressive multiple sclerosis involves follicular T-helper, Th17- and activated B-cells and correlates with progression. PloS One (2013) 8(3):e57820. 10.1371/journal.pone.0057820 23469245 PMC3585852

[51] Liu D Liu J Wang J Guo L Liu C Jiang Y. Distribution of circulating T follicular helper cell subsets is altered in immunoglobulin A vasculitis in children. PloS One (2017) 12(12):e0189133. 10.1371/journal.pone.0189133 29236760 PMC5728569

[52] Fonseca VR Graca L. Contribution of FoxP3+ Tfr cells to overall human blood CXCR5+ T cells. Clin Exp Immunol (2019) 195(3):302–4. 10.1111/cei.13245 PMC6378382 30632146

[53] Tipton C Fucile C Darce J Chida A Ichikawa T Gregoretti I. Diversity, cellular origin and autoreactivity of antibody-secreting cell expansions in acute Systemic Lupus Erythematosus. Nat Immunol (2015) 16(7):755–65. 10.1038/ni.3175 PMC4512288 26006014

[54] Hansen A Reiter K Ziprian T Jacobi A Hoffmann A Gosemann M. Dysregulation of chemokine receptor expression and function by B cells of patients with primary Sjögren’s syndrome. Arthritis Rheum (2005) 52:2109–19. 10.1002/art.21129 15986367

[55] Hansen A Odendahl M Reiter K Jacobi A Feist E Scholze J. Diminished peripheral blood memory B cells and accumulation of memory B cells in the salivary glands of patients with Sjögren’s syndrome. Arthritis Rheumatol (2002) 46(8):2160–71. 10.1002/art.10445 12209521

[56] Cornec D Saraux A Pers J Jousse-Joulin S Marhadour T Roguedas-Contios A. Diagnostic accuracy of blood B-cell subset profiling and autoimmunity markers in Sjögren’s syndrome. Arthritis Res Ther (2014) 16(1):R15. 10.1186/ar4442 24433480 PMC3978459

[57] Rodríguez-Bayona B Ramos-Amaya A Pérez-Venegas J Rodríguez C Brieva J. Decreased frequency and activated phenotype of blood CD27 IgD IgM B lymphocytes is a permanent abnormality in systemic lupus erythematosus patients. Arthritis Res Ther (2010) 12(3):R108. 10.1186/ar3042 20525218 PMC2911899

[58] Barone F Bombardieri M Rosado MM Morgan PR Challacombe SJ De Vita S. CXCL13, CCL21, and CXCL12 expression in salivary glands of patients with Sjogren’s syndrome and MALT lymphoma: association with reactive and malignant areas of lymphoid organization. J Immunol (2008) 180(7):5130–40. 10.4049/jimmunol.180.7.5130 18354239

[59] Fonseca V Agua-Doce A Maceiras A Pierson W Ribeiro F Romão V. Human blood Tfr cells are indicators of ongoing humoral activity not fully licensed with suppressive function. Sci Immunol (2017) 2(14):pii: eaan1487. 10.1126/sciimmunol.aan1487 PMC7116402 28802258

[60] Wing JB Kitagawa Y Locci M Hume H Tay C Morita T. A distinct subpopulation of CD25- T-follicular regulatory cells localizes in the germinal centers. Proc Natl Acad Sci U.S.A. (2017) 114(31):E6400–E9. 10.1073/pnas.1705551114 PMC5547636 28698369

[61] Ding Y Li J Yang P Luo B Wu Q Zajac A. Interleukin-21 Promotes Germinal Center Reaction by Skewing the Follicular Regulatory T Cell to Follicular Helper T Cell Balance in Autoimmune BXD2 Mice. Arthritis Rheumatol (2014) 66(9):2601–12. 10.1002/art.38735 PMC4146687 24909430

[62] Jandl C Liu S Cañete P Warren J Hughes W Vogelzang A. IL-21 restricts T follicular regulatory T cell proliferation through Bcl-6 mediated inhibition of responsiveness to IL-2. Nat Commun (2017) 8:14647. 10.1038/ncomms14647 28303891 PMC5357862

[63] Cicalese M Gerosa J Baronio M Montin D Licciardi F Soresina A. Circulating Follicular Helper and Follicular Regulatory T Cells Are Severely Compromised in Human CD40 Deficiency: A Case Report. Front Immunol (2018) 9:1761. 10.3389/fimmu.2018.01761 30131802 PMC6090258

[64] Ohlsson M Szodoray P Loro LL Johannessen AC Jonsson R. CD40, CD154, Bax and Bcl-2 expression in Sjögren’s syndrome salivary glands: a putative anti-apoptotic role during its effector phases. Scand J Immunol (2002) 56(6):561–71. 10.1046/j.1365-3083.2002.01168.x 12472667

[65] Roescher N Lodde BM Vosters JL Tak PP Catalan MA Illei GG. Temporal changes in salivary glands of non-obese diabetic mice as a model for Sjögren’s syndrome. Oral Dis (2012) 18(1):96–106. 10.1111/j.1601-0825.2011.01852.x 21914088 PMC3435870

[66] Spolski R Leonard WJ. Interleukin-21: a double-edged sword with therapeutic potential. Nat Rev Drug Discovery (2014) 13(5):379–95. 10.1038/nrd4296 24751819

[67] Voynova E Mahmoud T Woods LT Weisman GA Ettinger R Braley-Mullen H. Requirement for CD40/CD40L Interactions for Development of Autoimmunity Differs Depending on Specific Checkpoint and Costimulatory Pathways. Immunohorizons (2018) 2(1):54–66. 10.4049/immunohorizons.1700069 30607385 PMC6309431

[68] Liu H Liu G Gong L Zhang Y Jiang G. Local suppression of IL-21 in submandibular glands retards the development of Sjögren’s syndrome in non-obese diabetic mice. J Oral Pathol Med (2012) 41(10):728–35. 10.1111/j.1600-0714.2012.01175.x 22643047

[69] Rao D Gurish M Marshall J Slowikowski K Fonseka C Liu Y. Pathologically expanded peripheral T helper cell subset drives B cells in rheumatoid arthritis. Nature (2017) 542(7639):110–4. 10.1038/nature20810 PMC5349321 28150777

[70] Espié P He Y Koo P Sickert D Dupuy C Chokoté E. First-in-human clinical trial to assess pharmacokinetics, pharmacodynamics, safety, and tolerability of iscalimab, an anti-CD40 monoclonal antibody. Am J Transpl (2020) 20(2):463–73. 10.1111/ajt.15661 31647605

[71] Fisher BA Szanto A Ng W-F Bombardieri M Posch MG Papas AS. Assessment of the anti-CD40 antibody iscalimab in patients with primary Sjögren’s syndrome: a multicentre, randomised, double-blind, placebo-controlled, proof-of-concept study. Lancet Rheumatol (2020) 2(3):e142–52. 10.1016/S2665-9913(19)30135-3 38263652

