# Supplementary material for: The Imbalance of Circulating Follicular T Helper Cell Subsets in Primary Sjögren’s Syndrome Associates With Serological Alterations and Abnormal B-Cell Distribution
Source: Front Immunol. 2021 Mar 19;12:639975. doi: 10.3389/fimmu.2021.639975 (PMC8018236; doi:10.3389/fimmu.2021.639975)
Supplement: Supplementary file 1 [file DataSheet_1.pdf]

Supplementary Material

The imbalance of circulating follicular T helper cell subsets in primary Sjögren's syndrome associates with serological alterations and abnormal B-cell distribution

Krisztina Szabó[1]*†, Ilona Jámbor[1]†, Antónia Szántó[1], Ildikó Fanny Horváth[1], Tünde Tarr[1], Britt Nakken[2], Peter Szodoray[2] and Gábor Papp[1]

[1]Division of Clinical Immunology, Faculty of Medicine, University of Debrecen, Debrecen, Hungary

[2]Department of Immunology, Oslo University Hospital, Rikshospitalet, and K.G. Jebsen Center for B cell malignancies, University of Oslo, Oslo, Norway

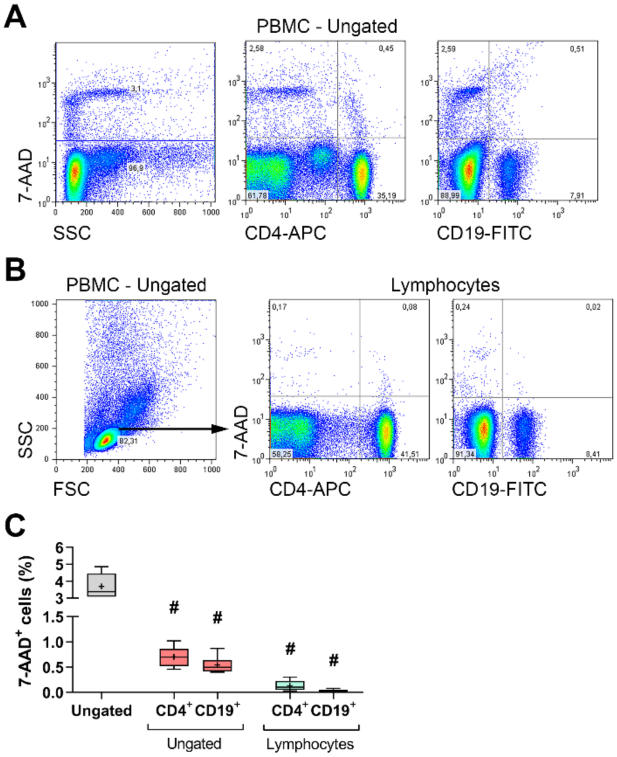

Supplementary Figure 1. Assessment of cell viability using 7-Aminoactinomycin D (7-AAD) staining in fresh PBMC samples. PBMCs were isolated from healthy controls (HC, n = 6) then cells were stained with anti-CD4-APC, anti-CD19-FITC fluorochrome-labelled antibodies and 7-AAD viability solution as described previously. Cells were assessed using FACS Calibur flow cytometer and further analyzed with FlowJo software. **(A)** Representative dot plots indicate the gating strategy and determination of viable cells within total PBMCs. **(B)** Representative dot plots indicate the gating strategy and determination of viable cells within lymphocytes. **(C)** Percentages of 7-AAD$^+$ cells, CD4$^+$7-AAD$^+$ cells and lymphocytes, CD19$^+$7-AAD$^+$ cells and lymphocytes. One-way ANOVA with Tukey's multiple comparisons test was used for data analysis. Box plots represent the interquartile range (IQR) with a line in the middle as median and "+" sign as the mean value. Statistically significant differences are indicated by # $p < 0.0001$.

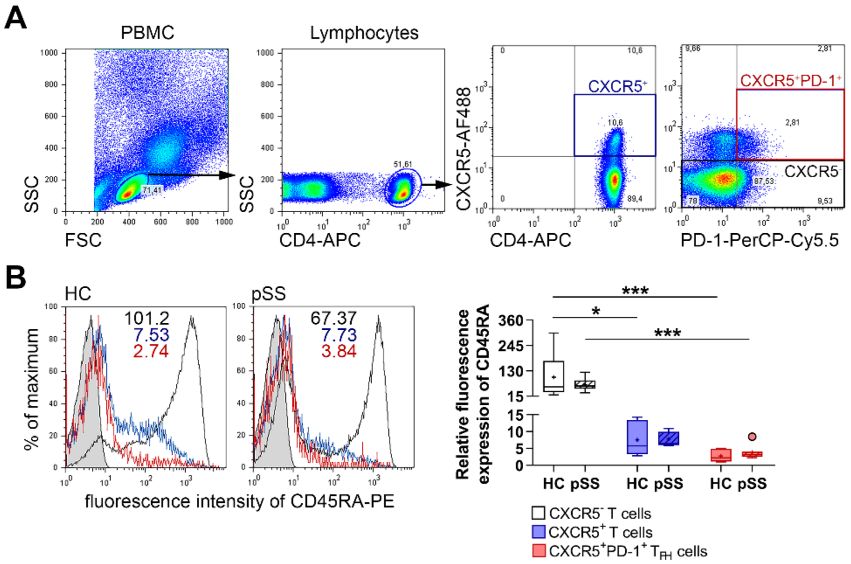

Supplementary Figure 2. Evaluation of the relative fluorescence expression of CD45RA on circulating CXCR5$^-$ T cells, CD4$^+$CXCR5$^+$ and CD4$^+$CXCR5$^+$PD-1$^+$ T cells. PBMCs were isolated from patients with pSS (n = 7) and healthy controls (HC, n = 6) then cells were stained with anti-CXCR5-AF488, anti-CD45RA-PE, anti-PD-1-PerCP-Cy5.5 and anti-CD4-APC fluorochrome-labelled monoclonal antibodies as described previously. Cells were analyzed using FACS Calibur flow cytometer and further examined with FlowJo software. **(A)** Representative dot plots show the gating strategy of T cell subsets. **(B)** Representative histograms display the expression of CD45RA in CXCR5$^-$ T cells (black), CD4$^+$CXCR5$^+$ (blue) and CD4$^+$CXCR5$^+$PD-1$^+$ (red) T cells. Relative fluorescence expression of CD45RA. Kruskal-Wallis test with Dunn's multiple comparisons test was used for statistical analysis. Box plots represent the interquartile range (IQR) with a line in the middle as median and “+” sign as the mean value. Statistically significant differences are indicated by *$p < 0.05$; ***$p < 0.001$.

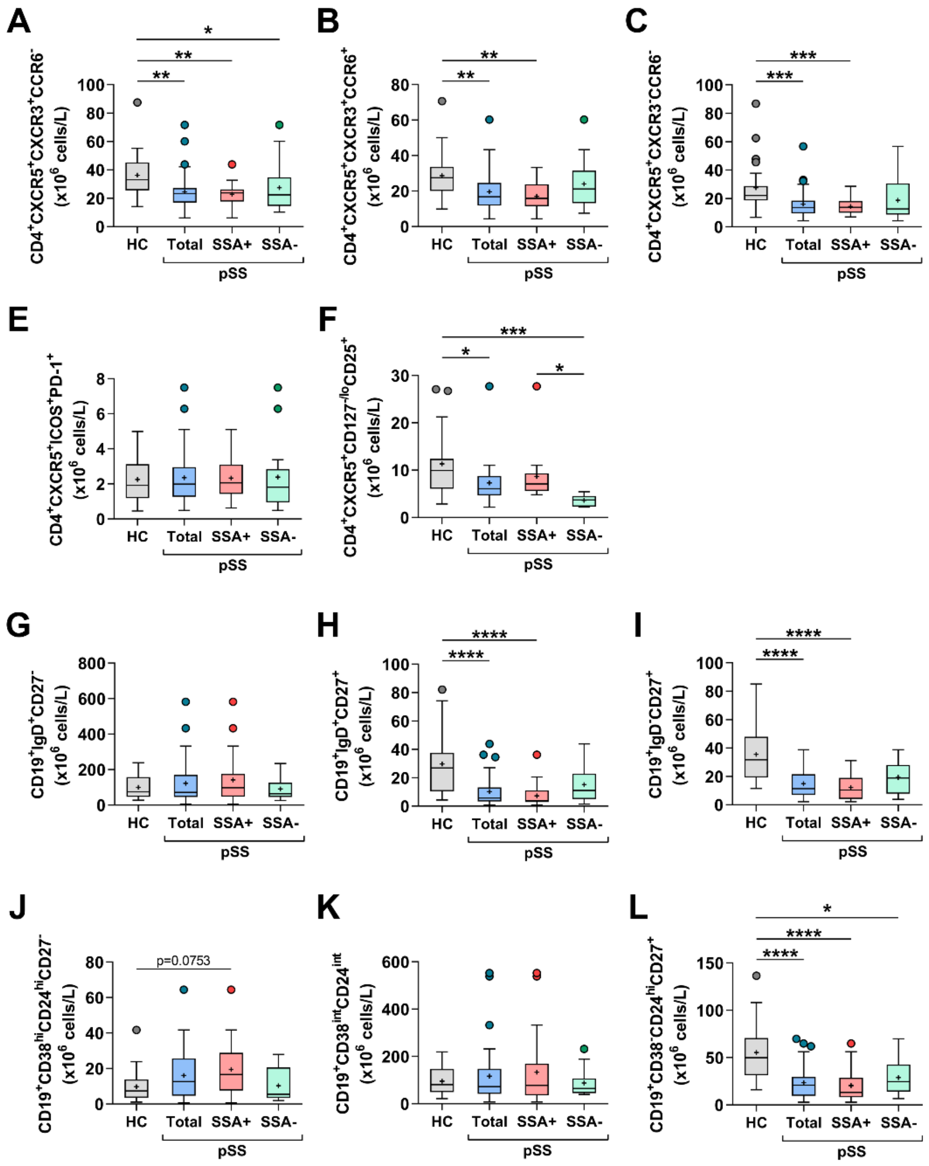

Supplementary Figure 3. Abnormal distribution of the absolute numbers of cT$_{\mathrm{FH}}$ subsets, cT$_{\mathrm{FR}}$ cells and different B cell subsets. The absolute numbers of **(A)** cT$_{\mathrm{FH}}$1, **(B)** cT$_{\mathrm{FH}}$1/17, **(C)** cT$_{\mathrm{FH}}$2, **(D)** cT$_{\mathrm{FH}}$17, **(E)** activated cT$_{\mathrm{FH}}$, **(F)** cT$_{\mathrm{FR}}$, **(G)** IgD^{+}CD27^{-} naive B cells, **(H)** IgD^{+}CD27^{+} non-switched memory B cells, **(I)** IgD^{-}CD27^{+} switched memory B cells, **(J)** CD38^{hi}CD24^{hi}CD27^{-} transitional B cells, **(K)** CD38^{+}CD24^{+} mature-naive B cells, **(L)** CD38^{-}CD24^{hi}CD27^{+} primarily memory B cells in pSS patients (n =38), pSS without anti-SSA(-) (n = 14) and pSS with anti-SSA(+) (n = 24) and healthy controls (HC; n=29). Data analysis were performed using one-way ANOVA with Tukey's multiple comparisons test or Kruskal-Wallis test with Dunn's multiple comparisons test. Correlation analysis was carried out with Spearman's test. Box plots represent the interquartile range (IQR) with a line in the middle as median and "+" sign as the mean value. Statistically significant differences are indicated by *$p < 0.05$; **$p < 0.01$; ***$p < 0.001$; ****$p < 0.0001$.

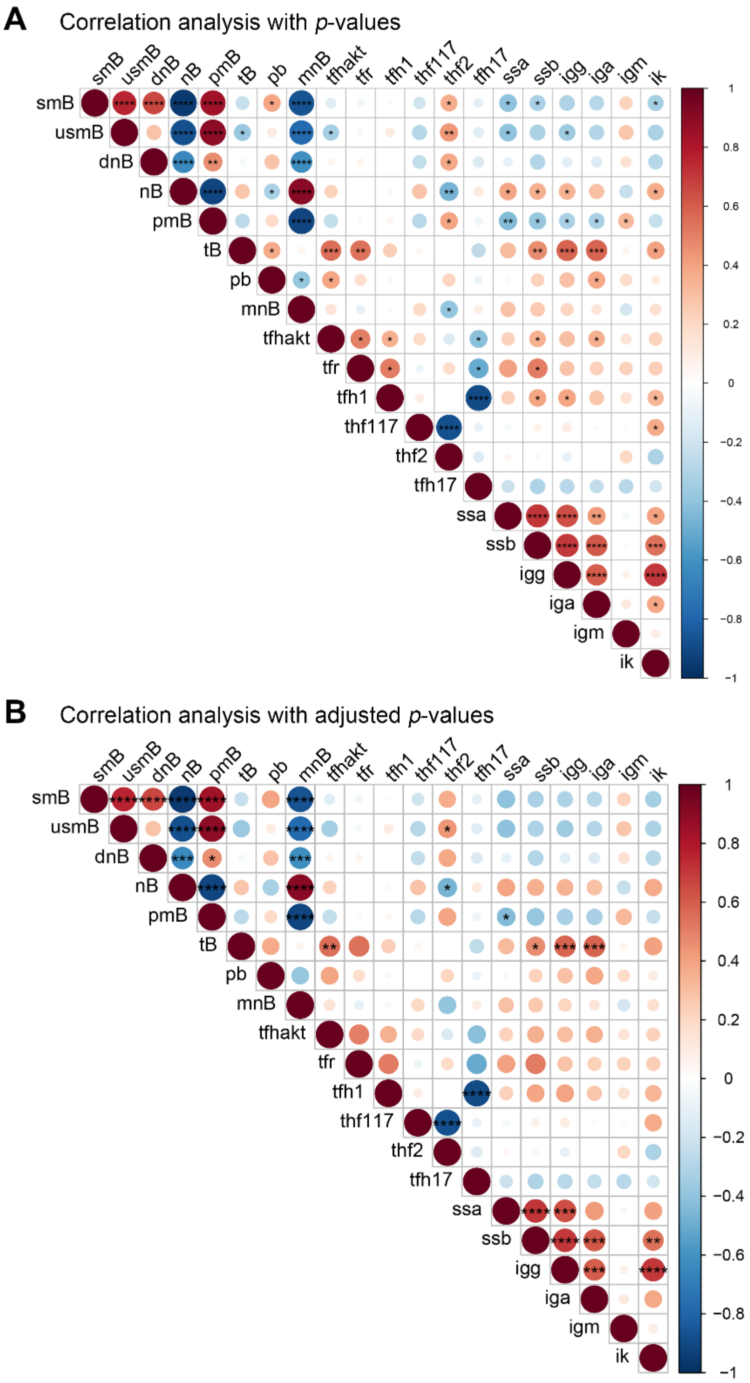

Supplementary Figure 4. Correlation analysis of the percentages of T_{FH} cell subsets, T_{FR} cells, B cell subsets and serological parameters in patients with pSS. Statistics and visualization of the correlation matrices were done with the R software, using the Hmisc and Corrplot packages. Spearman's correlation analysis were used for the correlations between the percentages of cell subsets and serological parameters and R-values were obtained using Corrplot function. **(A)** Correlation analysis with *p*-values and **(B)** adjusted *p*-values.
